# The influence of partial sleep restriction on repeated sprint ability and reaction time in university athletes

**DOI:** 10.3389/fspor.2025.1519987

**Published:** 2025-09-11

**Authors:** Benjamin R. Wannell, Felix M. J. Brunner, Zoe Lovibond, Bert O. Bond

**Affiliations:** Public Health and Sport Sciences, Faculty of Health and Life Sciences, University of Exeter, Exeter, United Kingdom

**Keywords:** sleep restriction, exercise performance, sprint interval exercise, university sport, sleep quality

## Abstract

**Introduction:**

Few studies have assessed the influence of acute sleep restriction on repeated sprint ability and reaction time, which are important characteristics of many sports. Additionally, no within-measures study has compared the acute effect of interrupting sleep to an equivalent quantity of sleep lost by going to bed late. This study examined the influence of sleep restriction, achieved by going to sleep late, or by interrupting sleep, on repeated sprint ability and reaction time.

**Methods:**

Sixteen university team sport players completed 3 conditions in a counterbalanced order; (1) normal sleep (“Control”), (2) 50% sleep loss achieved by going to be late (“Late”), and (3) 50% sleep loss achieved by waking in the middle of their scheduled sleep (“Interrupt”). The following morning, participants completed ten, 8 s all-out cycle sprints, each separated by 52 s recovery. Reaction time to a Go/No-Go test was measured during each recovery interval.

**Results:**

Peak and minimum power output for each repeated sprint interval was always lower in the Late [mean difference (MD) -17 W, *P* = 0.014, and MD -14 W, *P* = 0.022] and Interrupt conditions (MD -25 W, *P* < 0.001 and MD -24 W, *P* < 0.001), compared to Control. Additionally, average power output was lower across all sprint attempts in Interrupt (MD -14 W, *P* = 0.007), but not Late (MD -7 W, *P* = 0.170) compared to Control. Reaction time was never different between conditions.

**Conclusion:**

One night of 50% sleep loss can acutely impair repeated sprint ability. Interrupting sleep might be more deleterious than an equivalent amount of sleep lost through late sleep onset.

## 1 Introduction

Sleep is a fundamental, restorative process required for physiological and cognitive functioning (1), and is thought to be particularly important for the recovery from high training loads or exertion in athletes (2). Interventions to promote total sleep duration are also associated with favourable outcomes in athletic groups (3), including improvements in endurance (4) and sprint (5) performance, and reaction time (5, 6). However, shortened sleep duration or sleep loss is common in athletic populations; the majority of athletes fail to routinely achieve their required sleep duration (7). The causes for this are multifactorial (8), but it is understood that pre-event anxiety (9, 10), travel (11) and disruption to typical sleep routines (12) can have an acute, deleterious influence on sleep the night before competition.

Inadequate sleep is understood to acutely impair athletic performance across a variety of exercise paradigms (13). However, our understanding regarding the effect of sleep loss is clouded by key methodological differences between studies, such as the duration of sleep loss (partial sleep restriction compared to complete sleep deprivation, where no sleep is achieved in at least 24 h), how performance is assessed, and the competitive level of the participants (2, 8, 14, 15). Furthermore, few studies have considered how sleep loss might influence the ability to perform repeated sprints, which is an important characteristic of many team sports (16, 17). Skein and colleagues demonstrated that 30 h of sleep deprivation impaired intermittent sprint performance in male team sport athletes (18), however such sleep loss has little ecological validity. In contrast, it has been demonstrated that restricting a night's sleep to just 4 h can reduce repeated sprint ability (19, 20), but not performance in the Yo-Yo Intermittent Recovery Test in Taekwondo players (21). Such sleep restriction has also been shown to impair reaction time and cognitive processing (22), which is an important determinant of performance for many sports.

Interestingly, a recent systematic review highlighted that the manner in which sleep is lost may be an important factor regarding exercise performance, rather than just sleep quantity *per se* (13). For example, partial sleep restriction caused by early awakening appears to be more detrimental than equivalent sleep loss due to late sleep onset, possibly due to an increased sense of sleepiness and lower arousal the following day (20, 22). There is therefore value in exploring the effect of common types of sleep loss. In consideration of this, the most frequently experienced cause of sleep loss is a difficulty in being able to fall asleep (9) – i.e., late sleep onset. However, this same study highlighted that one third of athletes may experience difficulties in staying sleep – i.e., waking during the night. Similarly, in a sample of elite Australian athletes who reported experiencing sleep loss the night before competition, 38% reported waking up in the middle of the night (23). Little is known about the effects this manner of sleep loss has on performance, and we are not aware of any study which has explicitly compared the effect of sleep interruption caused by waking during the night against another type of sleep loss. This research gap is also of interest given our understanding that sleep is a dynamic process involving several ∼90 min “cycles” of different sleep stages (2). These stages have distinct regulatory and restorative roles which may have relevance to an athlete, for example the control of testosterone (24), growth hormone and cortisol (25), along with memory and skill consolidation (26). A disruption to this natural progression and completion of sleep cycles (i.e., through mid-night awakening) presents a qualitatively different challenge than the same duration of sleep restriction achieved through difficulty in falling asleep.

Given the above, the purpose of this study was to examine the influence of sleep restriction, achieved through going to sleep late, or by interrupting sleep, on repeated sprint ability and reaction time in university student athletes. It was hypothesised that repeated sprint performance would be impaired following both sleep restriction protocols, and that interrupting sleep in the middle of the night might be more adverse for performance than late sleep onset.

## 2 Methods

### 2.1 Study design

Ethical approval for the study was granted by the institutional research ethics committee. The exclusion criteria for the study included any current contraindications to exercise, the presence of any medical condition which might be exacerbated by sleep restriction, or any reported issues in habitual sleep patterns (i.e., shift work/irregular sleep patterns, sleep apnoea, failure to routinely achieve 7–9 h of sleep per night, or any self-declared issues with falling asleep or staying asleep). Eighteen university students who competitively played a team sport for the University of Exeter (football, lacrosse, rugby union) provided written informed consent to take part. However, 2 participants withdrew due to injury (unrelated to the study) during the data collection period. Thus, data are presented for 16 participants (8 male; 19.9 ± 1.9 years, body mass index 23.5 ± 1.8 kg/m^2^). Participants habitual sleep duration was 8.9 ± 0.5 h per night, with a mean global Pittsburgh Sleep Quality Index score of 5 ± 3 out of 21. Five participants were identified as “poor sleepers” using the Pittsburgh Sleep Quality Index cut off value of >5 (27).

**Figure 1:**
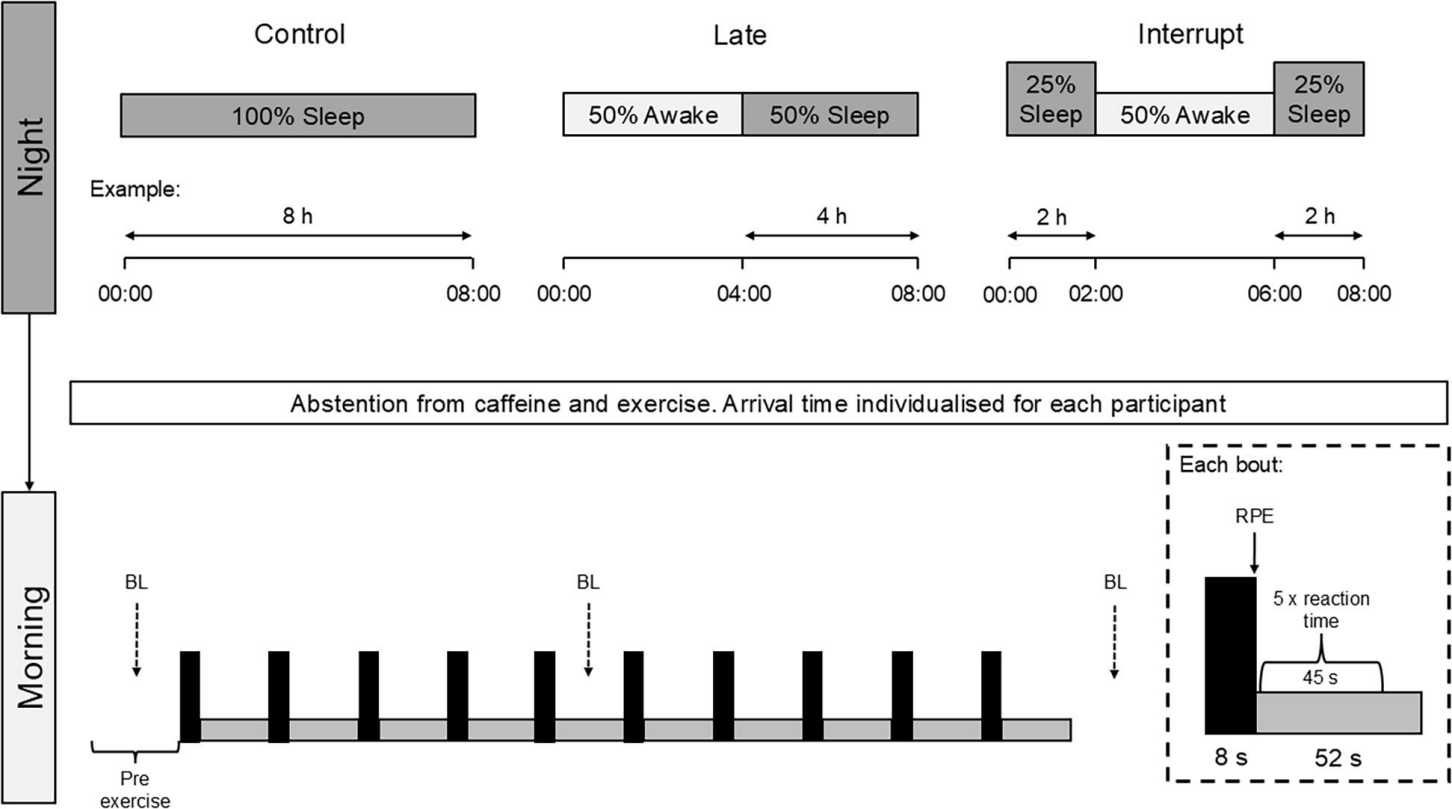
Protocol schematic. An example amount of sleep is provided for each condition, with a participant habitually going to bed at midnight and rising at 08:00. The 8 s all-out sprint and 52 s recovery (unloaded pedalling) is described by the black and grey rectangles in the lower pane, respectively. The dashed arrows represent the collection of fingertip capillary blood samples for blood lactate (BL) analysis before exercise, after the fifth sprint and 1 min after the final sprint. RPE, rating of perceived exertion as determined using the Borg 6–20 scale.

Participants were initially familiarised to all procedures, and then reported to the laboratory on three separate occasions, as described in Figure 1. Repeated sprint ability and reaction time were determined the morning after (1) a normal night of sleep (Control), (2) 50% sleep restriction achieved by going to bed late (Late), and (3) 50% sleep restriction by going to bed at the normal time, but then awakening, before returning to sleep (Interrupt). The precise duration of the three sleep protocols, and the timings of the 50% sleep restriction in the Late and Interrupt trials, was individually calculated according to the habitual routine of each participant. The three conditions were performed in a counterbalanced order and were separated by a minimum of 5 days (mean 9 ± 2 days). Participants were required to email a member of the research team every 30 min during the night of the Late and Interrupt conditions in order to confirm adherence to the sleep restriction protocols. This was also verbally confirmed by the participants upon arrival to the laboratory. Laboratory visits were rescheduled if a participant was unable to achieve the intended amount of sleep (i.e., a poor night's sleep in the Control condition, or inability to go back to sleep in the Interrupt condition). Participants always slept at home, in line with their normal routine apart from the nature of the sleep restriction trials. Participants were instructed not to consume anything (apart from water) during all conditions after their time of going to bed in the Control condition, and then to replicate their habitual breakfast in the morning prior to reporting to the laboratory. The precise start time of the morning visit was negotiated with each individual participant in order to prevent any shortening of typical wake times. This start time was then kept consistent for each participant, and ranged from 08:15 to 09:30 for the group.

Participants performed ten, 8 s all-out cycle sprints against a resistance of 8.5% body mass on a friction-braked cycle ergometer (Monark 849e, Vansbro, Sweden). Repeated sprint ability is considered to be an integral demand of many invasion-game sports. An 8 s sprint duration was specifically selected as it approximately reflects the time taken to perform repeated 20 m sprint (running) challenges, which have been shown to be a predictor of football (soccer) match performance (16). Each sprint was performed under strong verbal encouragement, and separated by 52 s of free wheel recovery. Rapid decision making is also an important component of many sports. Accordingly, our study incorporated the Go/NoGo test during the sprint exercise protocol, in order to consider whether the ability to quickly process information and react when under exertion is altered by acute sleep restriction. Specifically, participants completed 5 repetitions of the Go/NoGo test by pressing a touch screen tablet in rapid response to the appearance of a green circle (“Go”) but not a green circle with a cross (“No-Go”), during the first 45 s of each recovery interval.

### 2.2 Study outcome measures

Each 8 s sprint was scrutinised for peak power, the time to peak power, minimum power and average power output. A fatigue index was calculated as the percentage reduction in power output during each 8 s sprint (i.e., the within-sprint bout difference between peak and minimum power output, expressed as a percentage of the peak power output). A fingertip capillary blood sample for [lactate] was taken immediately before the first sprint, approximately 30 s after the fifth sprint, and 60 s after the final (tenth) sprint. Blood [lactate] was immediately analysed in whole blood using a Biosen C-Line Analyser (EKF Diagnostics, Germany). The Borg 6–20 scale (28) was also used to determine rating of perceived exertion (RPE) at the end of each 8 s sprint. Reaction time during each recovery interval was calculated as the mean of the response times to each “Go” stimulus.

### 2.3 Statistical analyses

Data were analysed using a linear mixed model with a random intercept (accounting for repeated measures within participants) plus fixed effects for condition (control/late/interrupt), and sprint number, and their interaction. Differences between conditions were interpreted using 95% confidence intervals (95% CI) of the mean difference (MD) and the *P* value. There was never an interaction effect for sex on any outcome, so data are pooled for men and women. Similarly, the use of a grouping variable failed to demonstrate that poor sleepers (i.e., those with a Pittsburgh Sleep Quality Index > 5) responded differently to the conditions compared to the rest of the population.

## 3 Results

**Figure 2:**
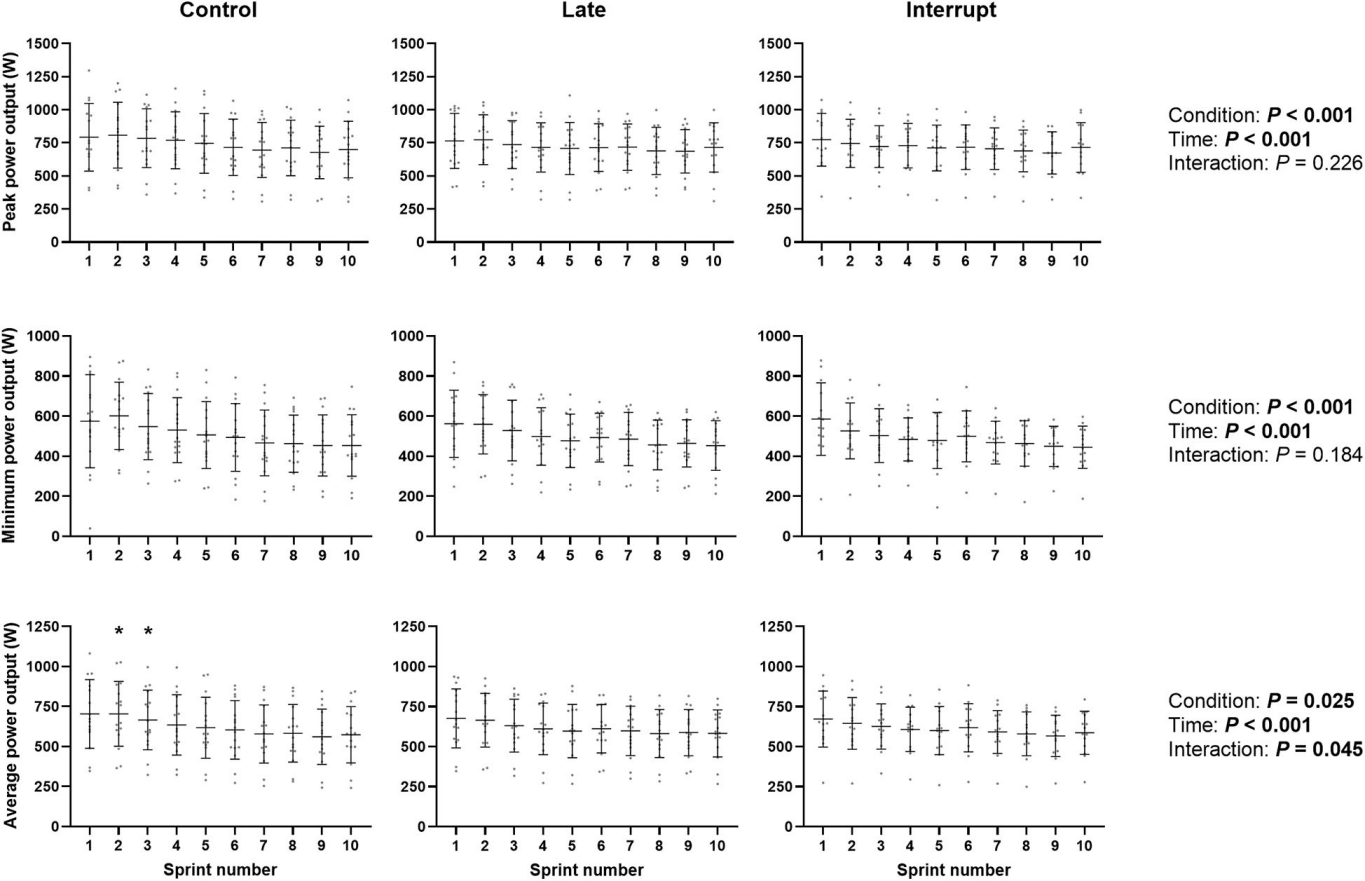
Repeated sprint ability. Data are presented as individual values, mean and standard deviation (error bars). Note the *y* axis scale is different for peak, minimum and average power output. * denotes a significantly greater average power output in the Control condition compared to both Late and Interrupt.

Repeated sprint ability data are presented in Figure 2. A main effect of condition (*P* < 0.001) was observed for peak power output. Compared to Control, peak power output was lower across all sprint attempts in Late (MD −17 W, 95% CI −30 to −3 W, *P* = 0.014) and Interrupt (MD −25 W, 95% CI −39 to −12 W, *P* < 0.001), with no differences between Late and Interrupt (MD 9 W, 95% CI −5 to 23 W, *P* = 0.209). However, there was no condition by sprint number interaction (*P* = 0.226).

A main effect of condition (*P* < 0.001), but not condition by sprint interaction (*P* = 0.184), was also observed for minimum power output. Compared to Control, minimum power was lower across all sprint attempts in Late (MD −14 W, 95% CI −25 to −2 W, *P* = 0.022) and Interrupt (MD −24 W, 95% CI −36 to −11 W, *P* < 0.001), with no differences between Late and Interrupt (MD 10 W, 95% CI −2 to 22 W, *P* = 0.117).

A main effect of condition (*P* = 0.025) and condition by sprint interaction (*P* = 0.045), was observed for the average power output of each 8 s all-out sprint. Compared to Control, average power output was lower across all sprint attempts in Interrupt (MD −14 W, 95% CI −24 to −4 W, *P* = 0.007), but not Late (MD −7 W, 95% CI −16 to 3 W, *P* = 0.170). There was no significant difference between Late and Interrupt (MD 7 W, 95% CI −3 to 18 W, *P* = 0.156). Follow up pairwise comparisons revealed that average power output was lower during sprint 2 (MD −39 W, 95% CI −69 to −9 W, *P* = 0.013) and 3 (MD −36 W, 95% CI −66 to −5 W, *P* = 0.023) in the Late condition compared to Control. Average power output was also lower during sprint 2 (MD −62 W, 95% CI −93 to −30 W, *P* < 0.001) and 3 (MD −43 W, 95% CI −74 to −11 W, *P* = 0.009) in the Interrupt condition compared to Control. There were no other differences between Control and Late (*P* > 0.085 for all) or Control and Interrupt (*P* > 0.068 for all). There were never any differences between Late and Interrupt (*P* > 0.162 for all).

There was no effect of condition, or condition by sprint interaction, for fatigue index (*P* = 0.543 and *P* = 0.938, respectively) or time to peak power (*P* = 0.070 and *P* = 0.806, respectively).

Blood lactate, RPE and reaction time responses to the repeated sprint cycling protocol are presented in Table 1. There was no effect of condition (*P* = 0.106), or condition by sprint interaction (*P* = 0.843), for blood lactate. A main effect of condition (*P* < 0.001) was observed for RPE. Compared to Control, RPE was always lower in Late (MD −1 AU, 95% CI −2 to −1, *P* < 0.001) and Interrupt (MD −1 AU, 95% CI −1 to 0, *P* < 0.001). RPE was also lower in Late compared to Interrupt (MD −1 AU, 95% CI −1 to 0, *P* < 0.001). There was no condition by sprint number interaction (*P* = 0.976).

**Table 1:** Physiological, perceptual and cognitive responses to repeated sprint cycling. Data are means and standard deviations. RPE, rating of perceived exertion (Borg 6–20 scale).

	Control	Late	Interrupt	Linear mixed model
Blood Lactate (mmol L^−1^)				
Pre exercise	1.71 ± 0.57	1.42 ± 0.34	1.44 ± 0.51	Condition: *P* = 0.106 Time: *P* < 0.001 Interaction: *P* = 0.843
Post sprint 5	9.00 ± 1.58	8.92 ± 1.45	8.21 ± 1.81	
Post sprint 10	11.22 ± 1.70	10.83 ± 1.82	10.66 ± 2.73	
RPE (AU)				
Post sprint 1	12 ± 3	10 ± 3	11 ± 3	Condition: *P* < 0.001 Time: *P* < 0.001 Interaction: *P* = 0.976
Post sprint 2	14 ± 2	12 ± 3	13 ± 3	
Post sprint 3	15 ± 2	14 ± 2	14 ± 3	
Post sprint 4	16 ± 2	15 ± 2	16 ± 2	
Post sprint 5	17 ± 2	16 ± 2	16 ± 2	
Post sprint 6	17 ± 2	16 ± 2	16 ± 2	
Post sprint 7	18 ± 2	17 ± 2	17 ± 2	
Post sprint 8	18 ± 1	18 ± 1	18 ± 1	
Post sprint 9	19 ± 1	18 ± 1	19 ± 1	
Post sprint 10	20 ± 1	19 ± 1	19 ± 1	
Reaction time (ms)				
Pre exercise	289 ± 31	297 ± 44	295 ± 35	Condition: *P* *=* 0.079 Time: *P* *=* 0.532 Interaction: *P* = 0.939
Post sprint 1	304 ± 42	307 ± 32	300 ± 47	
Post sprint 2	303 ± 36	298 ± 40	293 ± 81	
Post sprint 3	306 ± 43	306 ± 69	284 ± 32	
Post sprint 4	311 ± 33	324 ± 78	300 ± 36	
Post sprint 5	312 ± 46	321 ± 78	301 ± 34	
Post sprint 6	316 ± 43	330 ± 122	302 ± 27	
Post sprint 7	302 ± 42	307 ± 49	309 ± 33	
Post sprint 8	303 ± 32	301 ± 42	317 ± 43	
Post sprint 9	296 ± 40	325 ± 52	298 ± 38	
Post sprint 10	305 ± 36	301 ± 46	301 ± 45	

Reaction time was never different between conditions (*P* = 0.079), with no condition by sprint number interaction effect (*P* = 0.939).

## 4 Discussion

The purpose of this study was to assess the acute effect of disrupted sleep on repeated sprint ability and reaction time in university student athletes, and to consider whether the manner in which sleep loss occurs is important. Our findings highlight that 50% sleep restriction, achieved either through going to bed late, or sleep interruption, had a small, detrimental effect on repeated sprint cycling ability. Additionally, average power output during these sprints was always lower following sleep interruption, but this was not the case following late sleep onset. Finally, we never observed any differences in reaction time during exercise.

Our observation that 50% sleep restriction has a small, deleterious effect on repeated sprint ability is consistent with other studies which documented an impairment in repeated sprint running the day after 4 h of sleep restriction (19, 20). These authors highlighted that unfavourable changes in mood or sleepiness might explain the performance impairment after partial sleep restriction. An interesting finding in our study was that RPE was always lower during exercise following sleep restriction – even though the exercise was designed to be of maximal effort. This finding is at odds with the increase in RPE reported during exercise following one night of sleep restriction (29), although that was only observed by these authors when morning sleep was restricted; no change in RPE was observed following evening sleep restriction (29). However, this is not a consistent finding (30). Indeed, a recent systematic review highlights that the RPE response to exercise following sleep perturbation is complex, and influenced by participant characteristics and the manner in which sleep loss is achieved (31). It is possible that the reduction in RPE observed in our study reflects a lower level of arousal, or readiness to exercise. For example, one night of sleep restriction has been shown to lower perceptions of vigour (19), and this might mean that participants were unable to knowingly give a maximal effort during the brief, all-out exercise protocol. The fact that participants are not able to be blinded to the condition means that a psychological effect underpinning this poorer performance cannot be discounted. Indeed, perceived sleep quality is a known predictor of subsequent performance (32), and sleep appears to be highly valued by athletes (33). It is possible that perceptions around the importance of sleep/sleep loss might lead to a self-fulfilling prophesy of impaired performance. However, any such psychological element is not able to fully explain what we know regarding sleep restriction and performance. For example, the deleterious effect of partial sleep restriction on repeated sprinting has been shown to be ablated following the consumption of caffeine, in a placebo-controlled study (19). Furthermore, a recent study reported that 40% sleep restriction acutely lowered the autonomic response to an exercise challenge (34), which catalyses the need for further mechanistic work in this area.

There are some important differences between our study design and others concerned with repeated sprint ability following a similar quantity of sleep loss (19, 20). Firstly, our exercise test was performed in the morning, rather than the afternoon (20) or evening (19) following the sleep intervention. This detail is important, as the effect of sleep restriction is reported to be less apparent in the morning (13, 20, 35), due to an increasing relative sleep debt throughout the day. This might explain why we did not observe any changes in reaction time, which have been reported later the following day (19), although this is not a universal finding (20, 36). With this in mind, reaction time has been shown to be impaired following 4 h of sleep restricted at the end of the night (i.e., early awakening) rather than late sleep onset (20). Importantly, this manner of sleep restriction has been shown to be more detrimental to repeated sprint performance than our approach of delaying sleep onset (20). Therefore, comparisons between these studies should be made with caution.

We restricted sleep onset time as this is the most common manifestation of sleep disturbance (9). However, a novelty of our work is the additional inclusion of a sleep interruption condition, which was matched to total sleep time lost, as this is also frequently reported in athletes (37). We observed that average power output during the repeated sprints was only lowered following sleep interruption. This further adds to a body of evidence highlighting the manner in which sleep loss occurs has an independent effect on subsequent performance (13). Whilst matching these two conditions for total sleep restriction is a conceptual strength of this study, it should be acknowledged that achieving 50% sleep loss through a continuous period of being awake during the middle of a night's sleep is unlikely to occur. Sleep fragmentation, whereby sleep is routinely disturbed through multiple, short awakenings, might hold greater ecological validity. Despite multiple awakenings and poor sleep quality being commonly reported by athletes (9, 37), a recent meta-analysis highlighted that very few data are available regarding the influence of sleep fragmentation on performance (35). Indeed, their systematic review of the literature only returned one such study, which used an extreme sleep disturbance protocol (repeated 7:13 min sleep-wake cycles) and only considered resting hormonal responses (24). We are aware of only a few interventional studies which considered the influence of sleep disruption (38, 39), but comparison between these studies is difficult due to differences in the amount of sleep lost, and the exercise challenges performed. We are also not aware of any study which compare sleep interruption or fragmentation to an alternative period of equal duration sleep loss. In this regard, our data help further the field, but the influence of this cause of sleep loss remains an interesting area for future research.

The present study adopted a within-measures design to consider the influence of two common types of sleep loss on a key parameter of team sport performance (repeated spring ability) in a target population. Despite these strengths, our investigation contains several limitations. Firstly, “sleep” is a dynamic process, and can be divided into specific sleep stages which are qualitatively different (40). We were unable to provide insight into the disruption to specific sleep stages, as these were not quantified, for example by polysomnography. Such an approach would be a valuable addition in future studies. However the use of such instrumentation can acutely alter sleep (12), and thus would require an extended familiarisation time which was beyond the scope of this study. Secondly, we did not account for preferred sleep/wake cycles or chronotype, which might plausibly interact with the manner of sleep restriction and the timing of assessing exercise performance (41) and could therefore have been controlled for (4). With regards to individual characteristics which might have influenced the effect of sleep restriction, it has recently been reported that men may be more vulnerable to autonomic disturbances during exercise after sleep restriction (34). We recruited an equal number of men and women, but did not observe any interaction effect of sex. However, we did not design our study to answer this question, which means that we are likely under-powered to explore any such interaction. We are also unable to extrapolate our findings beyond the specific repeated sprint cycling protocol used, as the acute influence of sleep restriction on exercise performance is dependent on the exercise domain (13). Finally, we were unable to deliver this study in a double-blind manner, but this methodological issue is germane for the field. Future work could consider a “nocebo” and placebo effect, for example by deliberately misleading participants regarding caffeine supplementation.

In conclusion, we observed that one night of 50% sleep loss has a small detrimental effect on repeated sprint performance, but not reaction time, the following morning. Additionally, we add to a growing body of evidence which demonstrates that the manner in which this sleep loss is achieved may have an independent effect on performance. Specifically, our data indicate that interrupting sleep might be more deleterious than an equivalent amount of sleep lost through late sleep onset. Therefore, the implementation of strategies to avoid mid-night awakening the night before competition (i.e., due to anxiety) in the 30%–40% of athletes who experience this phenomenon might be beneficial. However, these differences were small and should now be confirmed with larger studies in the future, preferably with simultaneous acquisition of sleep architecture data.

## Data Availability

The raw data supporting the conclusions of this article will be made available by the authors, without undue reservation.
